# Web-Based Scaffolds: The Feasibility of a Constructivist Approach to Oncology Fellow Learning

**DOI:** 10.2196/52501

**Published:** 2024-02-23

**Authors:** Sam Brondfield, Matthew Schwede, Tyler P Johnson, Shagun Arora

**Affiliations:** 1 Department of Medicine University of California, San Francisco San Francisco, CA United States; 2 Department of Medicine Stanford University Palo Alto, CA United States

**Keywords:** constructivist learning, scaffolded learning, graduate medical education, fellowship training, oncology, feasibility, medical education, pilot study, study, online learning, online tool, online tools, remote learning, e-learning, training, cancer

## Abstract

In this 2-institution feasibility pilot, oncology fellows used and updated freely available web-based learning tools (scaffolds) in a constructivist fashion.

## Introduction

Succinct and updated oncology fellow learning materials are lacking. Additionally, fellow didactic learning often takes the form of passive lectures, which is undesirable [1,2]. Constructivist learning, wherein learners construct their own knowledge, is rare for fellows.

We piloted “scaffolds”—succinct slide sets shared across oncology trainees—and evaluated feasibility [3,4]. Throughout training, fellows can update the shared scaffolds in a constructivist fashion, thereby providing updated resources for themselves and colleagues.

## Methods

### Study Design

Two institutions participated—University of California, San Francisco (UCSF), and Stanford University. From 2018 to 2019, SB—a UCSF oncologist—designed 12 scaffolds, using Google Slides covering the solid tumor chapters from the American Society of Clinical Oncology’s Self-Evaluation Program (ASCO-SEP) textbook [5]. Hematology, gynecologic oncology, and neuro-oncology were omitted for this pilot. Scaffolds included text and images synthesized from ASCO-SEP and National Comprehensive Cancer Center guidelines. For brevity, the slides instructed fellows to adhere to length limits when making edits.

We emailed scaffold links to all first- to third-year UCSF (n=21) and Stanford University (n=27) oncology fellows in July 2019 and July 2020. Use was optional, and fellows could access and update the scaffolds anonymously at any time. Updates were audited by SB.

In December 2021, to evaluate feasibility outcomes (*fidelity*: degree to which the innovation was implemented as intended; *appropriateness*: perceived fit of the innovation; *self-efficacy*: belief in the ability to execute the innovation’s goals) [6], we reviewed updates tracked in Google Slides and conducted 2 voluntary feedback focus groups (UCSF: facilitated by SB; Stanford University: facilitated by MS—a Stanford University oncology fellow) with 4 fellows each. Focus group size was determined by responses to recruitment emails. Consent and demographic information were obtained. Participants did not need to use the scaffolds, as we were also exploring barriers to use. Focus groups were recorded and professionally transcribed. SB and MS independently reviewed the transcripts and generated themes through iterative discussion [7].

The scaffolds were updated in 2023 by SB (available on Google Drive) [8].

### Ethical Considerations

UCSF and Stanford University institutional review boards granted exemption (#20-31645) and approval (#57766), respectively. Participants received an information sheet and verbally consented before each focus group. Transcripts omitted personal identifiers, and interviewers never revealed participant identities to the rest of the study team. Participants received a US $10 electronic gift card.

## Results

### Fidelity

From July 2019 to December 2021, fellows made 60 updates (Table 1), ranging from new trials to changes in management; none were erroneous. SB made 9 edits for brevity.

**Table 1:** Number of updates to solid oncology scaffolds during the pilot period (July 2019 to December 2021).

Scaffolds	Updates by fellows (N=60), n	Updates by auditor (N=9), n
Bladder/kidney/adrenal	1	1
Breast	17	0
Gastrointestinal (lower)	5	0
Gastrointestinal (upper)	9	1
Germ cell	2	2
Head/neck	1	0
Lung (nonsmall cell)	3	1
Lung (small cell/other thoracic)	1	1
Melanoma	1	1
Prostate	6	0
Salivary/thyroid	2	1
Sarcoma	12	1

### Appropriateness

Focus group participants (N=8) were women and included Asian (n=3, 37.5%), White (n=3, 37.5%), Black (n=1, 12.5%), mixed-race (n=2, 25%), first-year (n=5, 62.5%), second-year (n=2, 25%), and third-year (n=1, 12.5%) fellows. Most (n=7, 87.5%) used the scaffolds. Qualitative analysis (Table 2) revealed that fellows felt the scaffolds were accessible and succinct learning tools, addressed the dearth of similar resources, served as effective preparation materials for clinical work and examinations, provided structured information for rapid reviews, and made interactions with complex resources easier.

**Table 2:** Qualitative analysis of transcripts from 2 oncology fellow focus groups (1 at the University of California, San Francisco, and 1 at Stanford University) that evaluated a pilot of solid oncology scaffolds (July 2019 to December 2021).

Theme		Supportive quotation
**Advantages**		
	Accessible, succinct resource	“[The scaffolds were] online and quickly accessible, for example on the shuttle on the way to work.”
	Addressed the dearth of similar resources	“There are few resources currently available for oncology fellows. [The scaffolds] filled a niche not currently filled by other resources.”
	Effective preparation materials for clinical work and examinations	“[The scaffolds] were a security blanket…helpful for clinic prep and inpatient consults.”
	Structured information for rapid reviews	“[The scaffolds] were helpful in that they provided frameworks…and approaches.”
	Easier subsequent use of more complex resources	“The guidelines felt less ‘foreign’ after reviewing the scaffolds…[the scaffolds] helped with knowledge retention from more complex resources.”
**Challenges**		
	Lack of fellow confidence in updating the scaffolds	“I wasn’t sure whether my learning points were important enough to add to the scaffold.”
	Lack of fellow ownership over the scaffolds	“I think fellows are probably less likely to update the scaffolds if they don’t feel responsible for them.”
	Too simple and broad to help with nuanced patient care	“Clinical care is so nuanced…the scaffolds may be too broad to help with some clinical situations.”
**Suggestions**		
	Improve visual appeal	“Maybe make them more visually appealing by including more figures or tables.”
	Clarify purpose and the fact that scaffolds can be updated	“I would make it clear that the slides are editable and that fellows should update them.”
	Facilitate opportunities for fellows to update scaffolds	“Asking fellows to update these might be good for their learning.”

### Self-Efficacy

Qualitative analysis revealed barriers to updating the scaffolds—fellows’ lack of ownership over the scaffolds and low confidence regarding appropriate updates.

## Discussion

### Principal Results

This pilot explored the feasibility of implementing constructivist scaffolds for oncology fellows. We found evidence of fidelity and appropriateness and delineated next steps to optimize self-efficacy. The scaffolds [8] can be downloaded and modified to avoid generating institution-specific scaffolds from scratch. To promote ownership and confidence, we recommend assigning fellows to update the scaffolds under faculty mentorship.

Despite demonstrating superior outcomes when compared to passive lectures, constructivist learning is rarely studied at the fellowship level [9-11]. We recommend evaluating constructivist learning modalities, such as scaffolds, in graduate medical education to enhance learning outcomes.

### Limitations

Though the focus groups suggested that multiple fellows used the scaffolds, Google Slides did not track how many fellows accessed or updated them. We did not incorporate multimedia components beyond images and tables (some needed to be removed before publication to respect copyright), nor did we include assessments in this pilot. We recommend that institutions consider incorporating multimedia content and assessments into the scaffolds. The number of focus group participants was small and not gender-diverse. Future studies should quantitatively evaluate usage patterns and user satisfaction to examine what factors drive utilization.

### Conclusion

We piloted a novel constructivist approach to fellow learning and found evidence of feasibility. Oncology educators may use and modify the scaffolds [8] to jump-start constructivist education for fellows at their institutions. Educators in other fields may wish to apply this model to their specialties.

## Abbreviations

ASCO-SEP: American Society of Clinical Oncology’s Self-Evaluation Program
UCSF: University of California, San Francisco

## Data Availability

The data sets analyzed during this study are not publicly available due to institutional review board restrictions but are available from the corresponding author on reasonable request.
